# Motor organisation of social play in children with autism

**DOI:** 10.1098/rsif.2025.0302

**Published:** 2025-11-12

**Authors:** Ruaridh Clark, Szu-Ching Lu, Anna Anzulewicz, Krzysiek Sobota, Lucy Thompson, Bibi Hagberg, Max Thorsson, Christos Tachtatzis, Ivan Andonovic, Alex McConnachie, Helen Minnis, Philip Wilson, Philip Rowe, Christopher Gillberg, Malcolm Macdonald, Jonathan Delafield-Butt

**Affiliations:** ^1^ Laboratory for Innovation in Autism, University of Strathclyde, Glasgow, Scotland, UK; ^2^ Department of Electronic and Electrical Engineering, University of Strathclyde, Glasgow, Scotland, UK; ^3^ Department of Computer and Information Science, University of Strathclyde, Glasgow, Scotland, UK; ^4^ Strathclyde Institute of Education, University of Strathclyde, Glasgow, Scotland, UK; ^5^ Department of Psychology, University of Warsaw, Warsaw, Poland; ^6^ Gilberg Neuropsychiatry Centre, University of Gothenburg, Gothenburg, Sweden; ^7^ Institute of Applied Health Sciences, University of Aberdeen, Aberdeen, Scotland, UK; ^8^ Robertson Centre for Biostatistics, University of Glasgow, Glasgow, Scotland, UK; ^9^ School of Health and Wellbeing, University of Glasgow, Glasgow, Scotland, UK; ^10^ General Practice, University of Copenhagen, Copenhagen, Denmark; ^11^ Department of Biomedical Engineering, University of Strathclyde, Glasgow, Scotland, UK

**Keywords:** autism, play, prospective motor control, child psychology

## Abstract

Play is a quintessential human behaviour, underpinned by motor organisation and fundamental for learning and development. However, the motor patterns underlying play have not been computationally characterised in children with autism, despite known play pattern differences, including reduced social and pretend play. Recent evidence of fundamental neuromotor disruption in autism suggests neuromotor organisation differences may underpin play differences. We employed a digital game to examine play patterns in 878 children aged 2.5–6 years old, including 372 diagnosed with autism spectrum disorders (ASD), 64 diagnosed with other neurodevelopmental disorders and 441 without known neurodevelopmental problems (WP). Computational characterisation of play patterns by network analysis revealed significant differences between groups in the motor organisation of its sequential steps. Children with ASD developed an indirect, two-step pattern during the social food-sharing aspect of the game, in contrast to a direct, single-step pattern by WP children. These findings provide new variables for the digital characterisation of ASD. They reveal differences in the sequential nature of goal-directed motor organisation made in play in autism that precede higher-order differences in social cognition and emotional regulation reported in the literature, giving important insight into the psychomotor nature of autism for its education, care and support.

## 1. Introduction

Young children with autism spectrum disorder (ASD) engage in play differently from their peers without neurodevelopmental problems (WP), displaying fewer and less varied play patterns [1–5]. They also engage in less spontaneous play than their WP counterparts or children with other neurodevelopmental disorders (OND) [3,4,6]. Play is an important learning experience, allowing children’s interests and intentions to test the world, explore its opportunities and learn its responses [7–9]. Reduction in play reduces learning opportunities with consequences for the development of cognitive and social skills [10,11], for learning from observing others’ play [4] and for generalising skills across domains [12].

Research into play in ASD has typically focused on symbolic aspects that afford the imaginative qualities of pretence or pretending [1–3]. Children with ASD have difficulties with symbolic play when compared with age-matched WP or OND children, and these difficulties are greater in free or spontaneous play [13]. However, functional play, where the play does not necessarily involve pretending, is also different in children with autism than that of their WP peers with less elaborated, less varied and less integrated play [5]. Object salience may overpower the functional and symbolic potential of play [14]. Children with autism produce more rigid, stereotyped play behaviours [15].

Extant research has neglected the motor organisation that underpins the structure of play. Play in children is produced by generative motor action organised by sequential, goal-directed motor steps to produce its playful outcomes. Each simple, single motor action generates new possibilities for future action [16–18]. These simple motor actions, such as reaching to grasp a block, become serially organised into complex sequences of actions or chains, such as reaching to grasp the block then stacking the block on another, to form more elaborate, complex play patterns in what Karl Lashley identified as the ‘serial organisation of behavior’ [19]. Such prospective action plans underpin the development of awareness, imagination and higher-order cognitive processes [16,20,21]. Sequential motor action and perception of others’ sequences afford social learning [22].

In children with autism, new evidence demonstrates a subtle, but significant disruption to prospective movement [23–28], and recent epidemiological analysis reveals 87% of children with autism are at risk, although rarely diagnosed, for co-morbid motor difficulties [29,30] that disrupt daily living skills [31]. In particular, the serial organisation of purposeful motor actions is disrupted in autism [32,33]. This suggests motor difficulties may underpin higher-order social cognition differences in autism, and the pretence that allows imaginative play [23]. Nevertheless, very little is known about the motor organisation of autistic play patterns and how they relate to levels of autistic impairment. In particular, the motor sequencing that makes up children’s playful projects as they translate into imaginative stories of interest and learning has not been studied.

**Figure 1.**
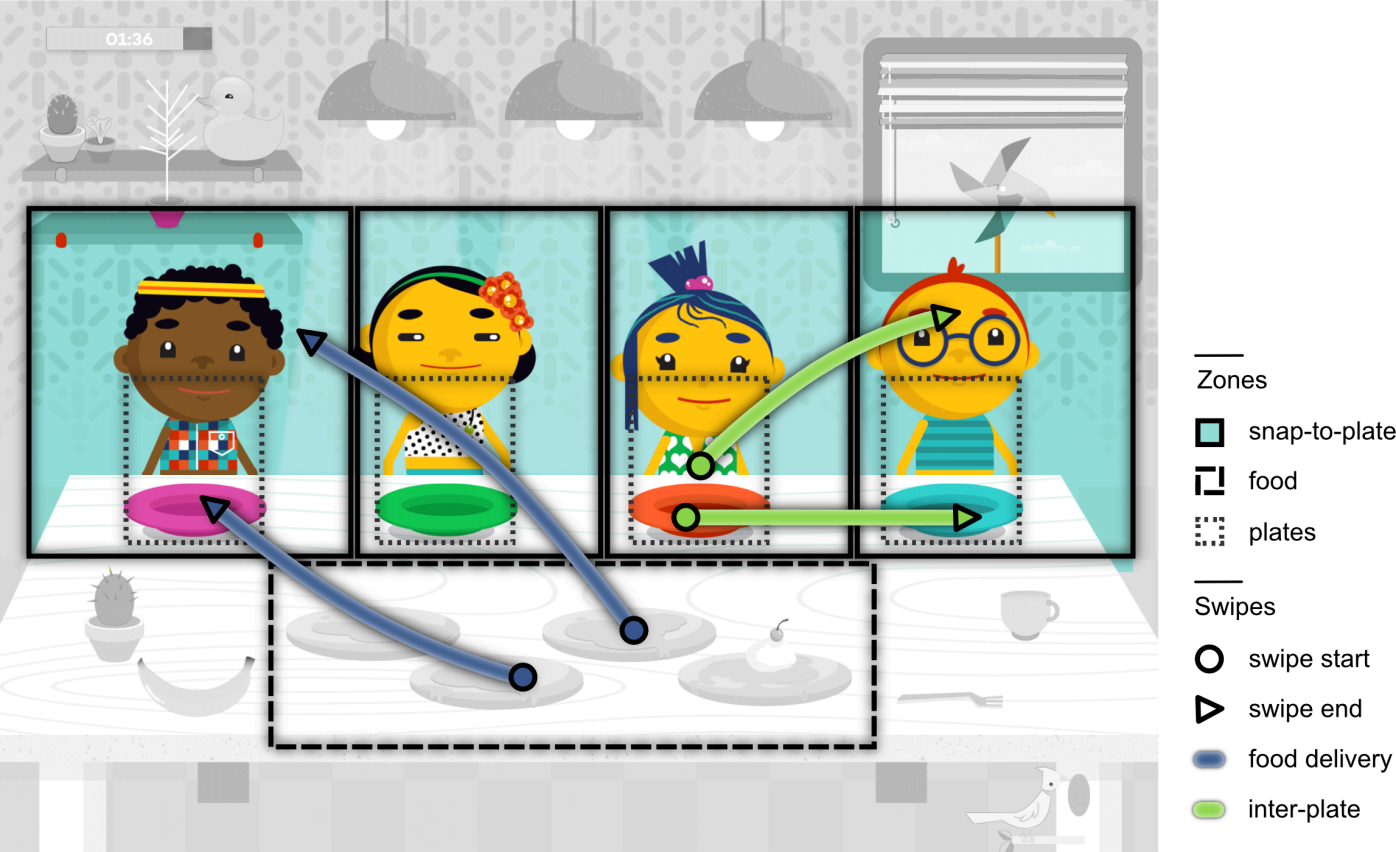
The serious game overlaid with the principal zones used to characterise swipe patterns. User swipes were categorised with reference to these zones; food delivery swipes originate in the food zone and end in a snap-to-plate/plate zone; delivered food is automatically placed on a plate or on top of any food items already present on a plate, with inter-plate swipes starting in a plate zone and ending in another snap-to-plate/plate zone.

In this study, we employed a serious iPad game for young children to computationally assess their play patterns in an ecologically valid manner (§5), and where the child was encouraged towards social aspects of gameplay (sharing food) with attractive sensory feedback (figure 1). Children were encouraged to drag four pieces of food from a serving area at the front (food zone) to deliver them to a set of four expectant children (snap-to-plate zones) to trigger feeding animations and an audible celebration. The interaction with social stimuli aligns with second-person neuroscientific approaches, which promote social interaction over observation due to the differing neural mechanisms involved [34]. Touch screen sensor data afforded computational analysis of gameplay through sequential motor patterns.

Gameplay was recorded by touch-screen data collection with continuous touch contacts tracked as swipe trajectories. The swipes were characterised by their origin and destination; those of relevance to social aspects of gameplay are illustrated in figure 1. The quantification of these swipes provides a basis for examining differences in gameplay. Conversion of all swipes that transitioned between zones into a single network, including both goal-directed and other gameplay swipes, enabled computational analysis of gameplay differences within the context of all gameplay swipe activity. The resultant network represents a user’s gameplay pattern, and computes the probabilities of transition between each zone. The first eigenvector of any swipe transition network indicates the principal direction of change. This is a measure of the incidence of zones as swipe destinations [35]. The incidence of plates as destinations, from locations in the game where food can be placed, provides insight into patterns of social food sharing in gameplay and reveals differences in those patterns between groups.

## 2. Results

### 2.1. Gameplay patterns between groups and across development

#### 2.1.1. Children with autism played with fewer movements

**Figure 2.**
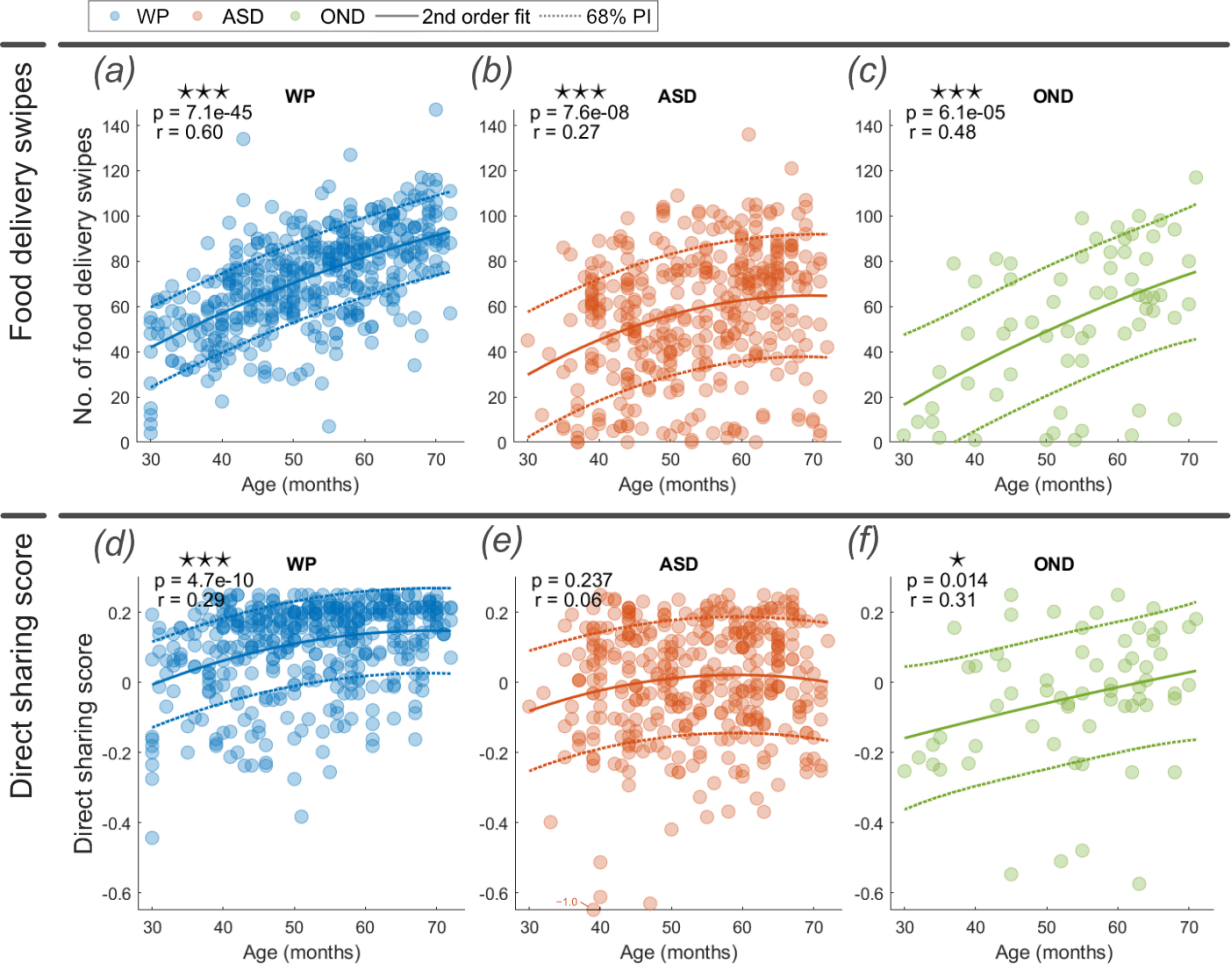
Number of food delivery swipes (*a–c*) and the direct sharing score (*d–f*) for WP, ASD and OND groups. The Spearman’s ranked correlation coefficient (*r*) and its significance (*p*) with age are reported at the top of each plot alongside the second-order fit line and illustrative 68% prediction interval. *Note*: ⋆ *p* < 0.05; ⋆⋆ *p* < 0.01; and ⋆⋆⋆ *p* < 0.001.

The game required moving four pieces of food from the foreground table to the empty plates in front of each character or to the characters themselves. These food delivery swipes were goal-directed actions that constituted the principal gameplay feature. The quantity of food delivery swipes increased by age in all groups (ASD, OND and WP), with significant positive correlations between age and the number of food delivery swipes (figure 2*a–c*). For the ASD group, the increase flattened notably after 50 months, suggestive of a ceiling effect that is in contrast to the continued increase in food delivery swipe activity in WP and OND groups.

Across all ages, children with ASD and OND completed significantly fewer food delivery swipes (median of 60 and 58.5, respectively) during the 5 min gameplay, in comparison with WP children (median of 74; *p* < 0.001 for both comparisons).

#### 2.1.2. Reduced development of direct food sharing in children with autism

A social pretence aspect of the game requires the food to be shared evenly across the four characters’ plates. To assess this social aspect of play within the context of other possible play actions (such as playing with other objects on the screen), we computed a ‘direct sharing score’ to measure the child’s prioritisation of food delivery swipes from the foreground food zone to all four of the plates/characters (‘direct food delivery swipes’) relative to gameplay swipes with other origin or destination zones (§5). This metric accounts for each gameplay swipe that transitions between zones, with a higher score indicating greater focus on evenly sharing food to the four characters—the standard expectation of sharing food in a social situation—with respect to other gameplay swipe activity.

Significant positive correlations between age and direct sharing score were observed in the WP and OND groups (figure 2*d*,*f*), demonstrating increasing adherence and interest to the expected norm; no correlation was observed in the ASD group (*p* = 0.237, figure 2*e*), despite an increase in food delivery swipes. Thus, the disposition to share food evenly with direct food delivery was greater and increased with age in WP and OND children, but not in ASD children.

Children with ASD and OND demonstrated significantly lower direct sharing scores than WP children (*p* < 0.001 for both comparisons), demonstrating reduced engagement in this social aspect as a proportion of total play.

#### 2.1.3. Children with autism developed an indirect approach to sharing

**Figure 3.**
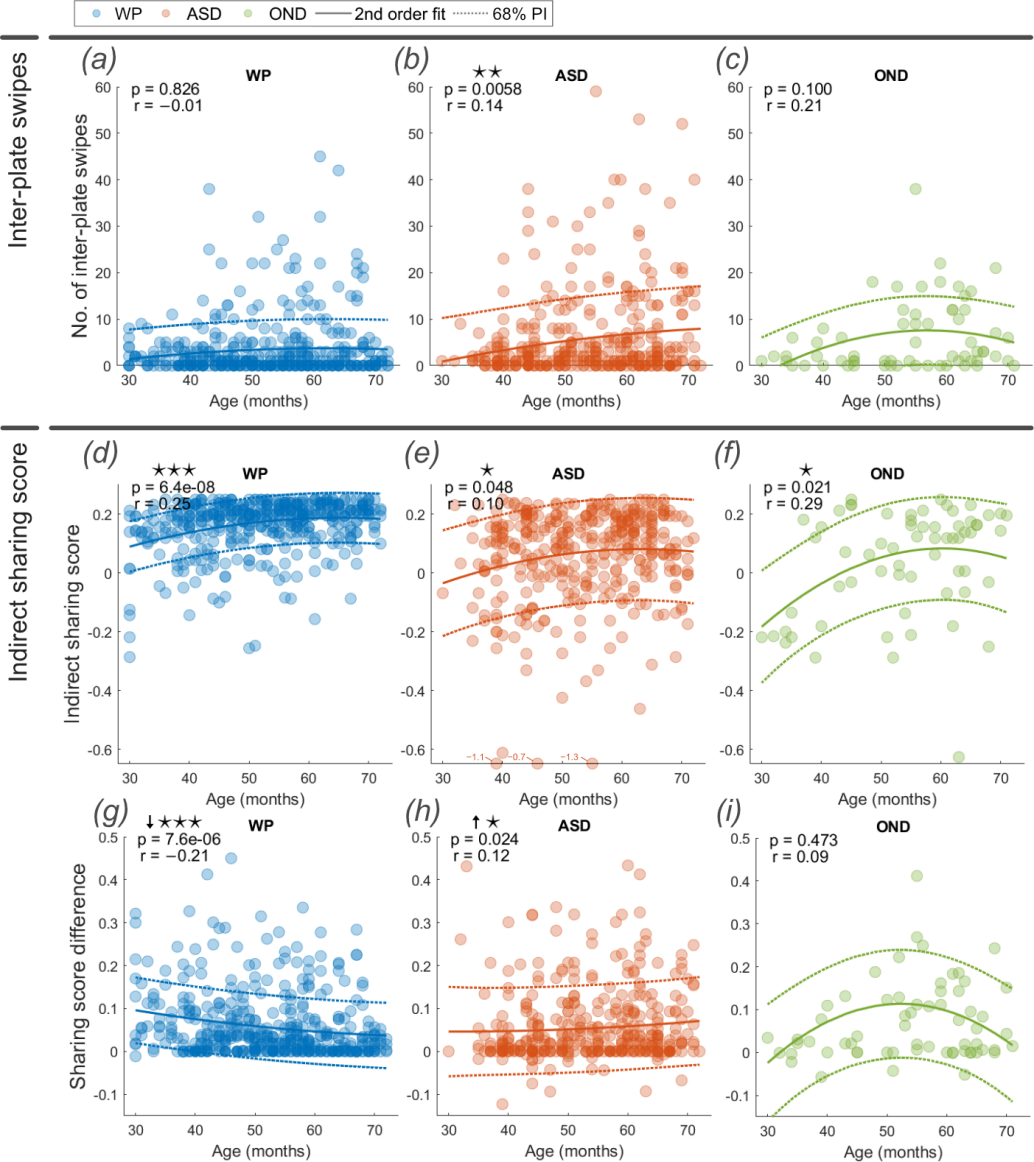
Number of inter-plate swipes (*a–c*), indirect sharing score (*d–f*) and sharing score difference, indirect – direct, (*g–i*) for WP, ASD and OND groups. The Spearman’s ranked correlation coefficient (*r*) and its significance (*p*) with age are reported at the top of each plot alongside the second-order fit line and illustrative 68% prediction interval. The boxplot comparisons detail significant differences evaluated using the Wilcoxon rank sum test. *Note*: ⋆ *p* < 0.05; ⋆⋆ *p* < 0.01; ⋆⋆⋆ *p* < 0.001.

In addition to directly delivering food pieces from the food zone to the characters’ plates, children may put more than one piece of food on the same plate and then redistribute them to another character’s plate. We call these ‘inter-plate swipes’ (figure 1). Interestingly, the ASD group demonstrated a significant positive correlation between age and the number of inter-plate swipes (*r* = 0.14, *p* < 0.01; figure 3*b*), in contrast to the WP and OND groups that did not (figure 3*a,c*). Furthermore, across all ages the ASD and OND groups displayed significantly higher numbers of inter-plate swipes than the WP group (*p* < 0.001 for both comparisons), altogether demonstrating preference for this gameplay form.

By categorising inter-plate swipes as food delivery swipes in the network, an ‘indirect sharing score’ was computed to account for both direct and indirect food delivery. Children with ASD demonstrated a significant positive correlation between age and indirect sharing score (*r* = 0.10, *p* < 0.05, figure 3*e*), despite a lack of development in the direct sharing score (figure 2*e*). In other words, children with ASD developed greater focus on sharing the food evenly, increasing with age, when we allow for both direct and indirect sharing strategies such as first stacking multiple pieces of food on the same plate and using inter-plate swipes to complete the sharing. The indirect sharing score, as with the direct score, is significantly lower in ASD than in WP (*p* < 0.001).

To better understand the development of indirect and direct sharing strategies, we calculated a ‘sharing score difference’ (indirect minus direct sharing score; figure 3*g–i*). A significant decrease in sharing score difference was observed in the WP group with age (*r* = −0.21, *p* < 0.001, figure 3*i*), while an increase in sharing score difference was observed in the ASD group as age increased (*r* = 0.12, *p* < 0.05, figure 3*j*). The increase shows that children with ASD, on average, developed an indirect route to the social aspect of sharing food evenly, rather than by the direct route alone (measured with the direct sharing score above), preference that developed in the WP group.

Altogether, these results demonstrate a prominence in autism of stacking food first, before distributing it evenly, rather than first distributing it evenly directly. The implication of this difference is profound, because it demonstrates a step-wise prospective progression in play in children with ASD, rather than one where the final goal is integrated from the start (§3) as is seen to develop in WP children (figure 3*i*).

### 2.2. Gameplay patterns stratified by autism spectrum disorder severity

#### 2.2.1. Number of food delivery swipes and sharing scores decreased with increased neurodevelopmental disruption

**Figure 4.**
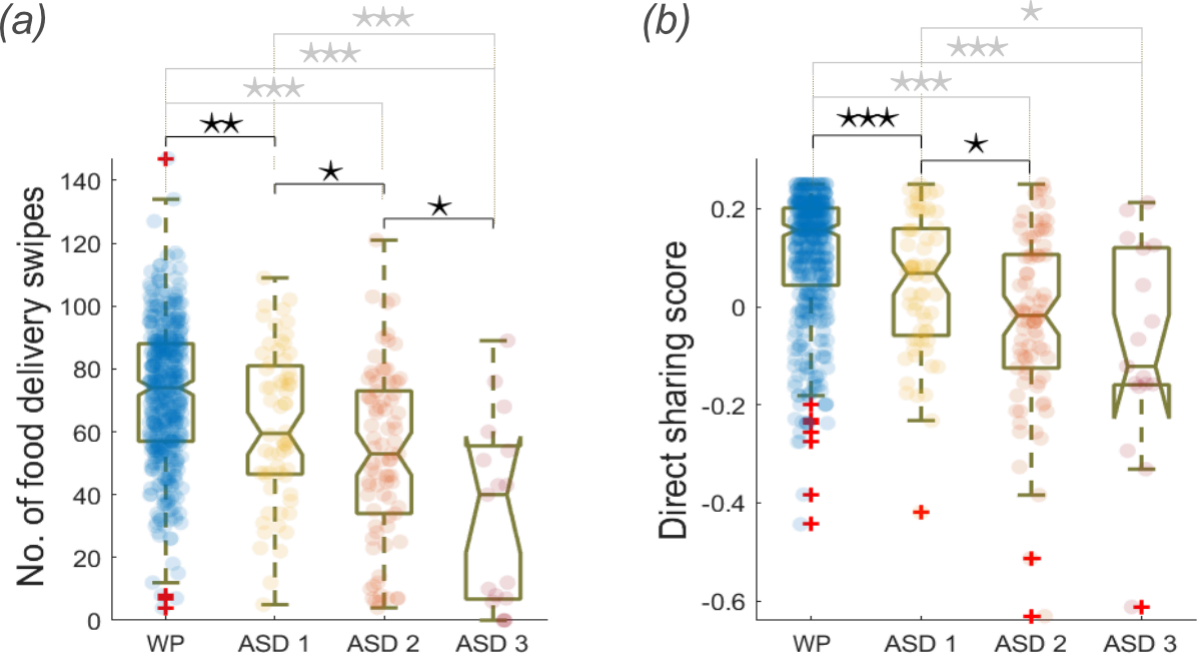
Number of food delivery swipes (*a*) and direct sharing scores (*b*) for WP and ASD children grouped by Severity Levels 1–3. For the boxplot comparison, significant differences between neighbouring boxes are evaluated using the Wilcoxon rank sum test. *Note*: ⋆ *p* < 0.05; ⋆⋆ *p* < 0.01; ⋆⋆⋆ *p* < 0.001.

Children in good neurodevelopmental health (WP) performed more food delivery swipes than children in the ASD and OND groups (figure 2*d*). Within the ASD group, food delivery swipes became increasingly prominent with increasing ASD functional severity level (figure 4*a*), as defined by DSM-5 criteria for daily support needs (§5). Similarly, direct sharing scores were significantly higher in the WP group compared with the ASD and OND groups (figure 2*h*). Within the ASD group, this feature became increasingly prominent with increasing ASD severity between levels 1 (minor severity) and 2 (moderate severity; *p* < 0.05; figure 4*b*). No significant difference was found between ASD severity levels 2 and 3 (*p* = 0.233). Indirect sharing scores produced similar differences in distribution (electronic supplementary material). Altogether, this suggests the group effect between WP and ASD children is exaggerated with exacerbated ASD severity.

### 2.3. Gameplay patterns stratified by sex

#### 2.3.1. Autism spectrum disorder male but not autism spectrum disorder female scores increased with age

**Figure 5.**
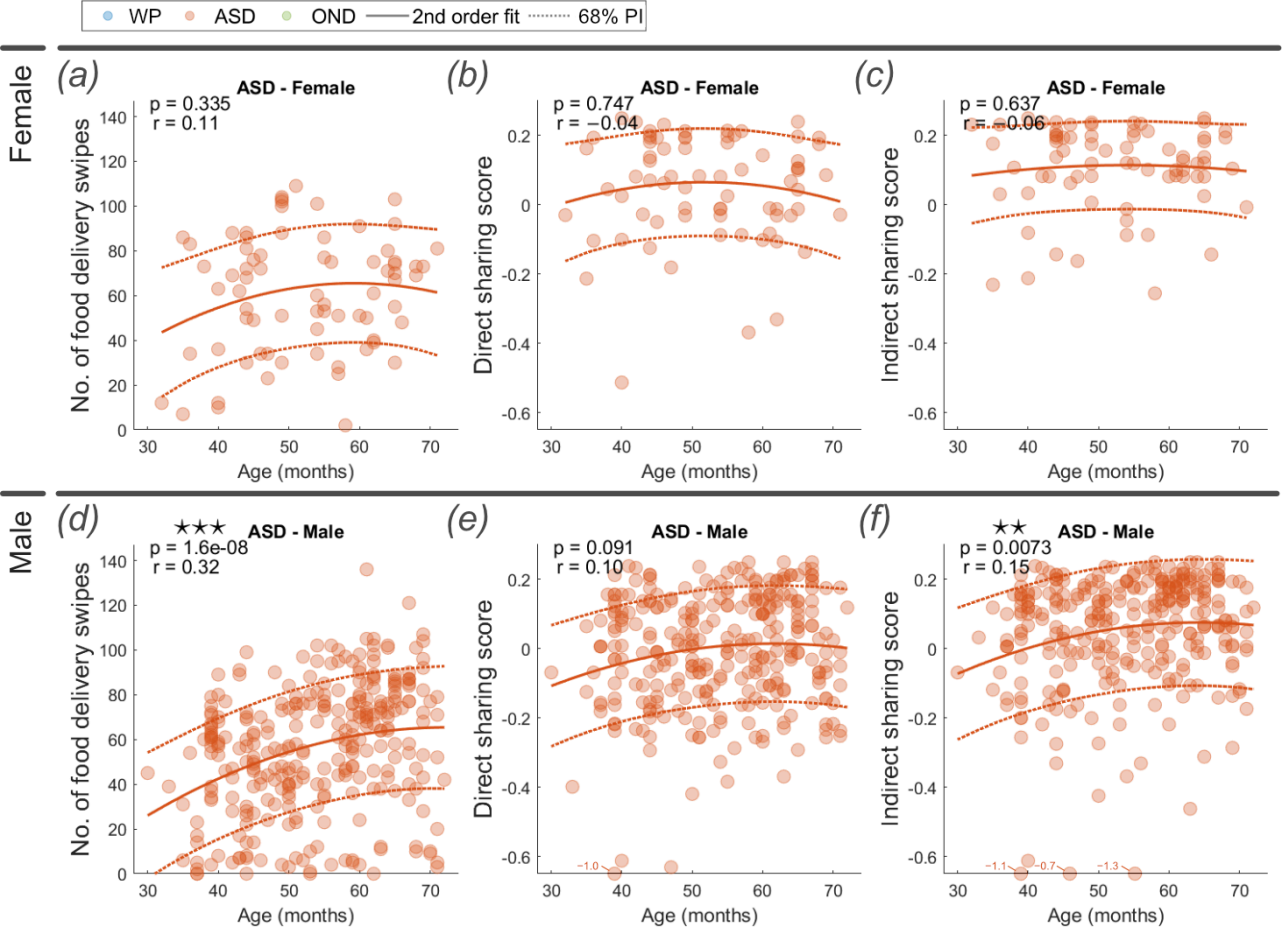
Number of food delivery swipes, direct sharing score and indirect sharing score for ASD group segregated by sex. The Spearman’s rank correlation coefficient (*r*) and its significance (*p*) are reported at the top of each plot with age, alongside the second-order fit line and illustrative 68% prediction interval. For the boxplot comparisons, significant differences are evaluated using the Wilcoxon rank sum test. *Note*: ⋆ *p* < 0.05; ⋆⋆ *p* < 0.01; ⋆⋆⋆ *p* < 0.001.

The ASD male group presented a significant positive increase by age in number of food delivery swipes (figure 5*d*, *r* = 0.32, *p* < 0.001) and indirect sharing score (figure 5*f*, *r* = 0.16, *p* < 0.001). In contrast, no significant change across children’s ages was observed within the ASD female group in number of food delivery swipes (figure 5*a*, *p* = 0.329), direct sharing score (figure 5*b*, *p* = 0.726) and indirect sharing score (figure 5*c*, *p* = 0.635). Both WP female and male groups followed similar trajectories across age, with significant positive increases in the number of food delivery swipes, and direct and indirect sharing scores (electronic supplementary material).

Despite no change across ages in the ASD female group, their direct and indirect sharing scores were significantly higher than those of the ASD male group (*p* < 0.05 and *p* < 0.01, respectively). A significantly higher direct and indirect sharing score was also noted for the WP female group over the WP male group (*p* < 0.001 for both sharing scores).

### 2.4. Socio-emotional, cognitive and behavioural differences

Scores for cooperativity, exposure to tablets, interest in tablet games and distractibility recorded on the day increased with age for WP, ASD and OND. Mood and arousal were also recorded but only found to increase with age for the OND group. None of these observation scores for socio-emotional status on the day of collection were found to correlate with the sharing scores.

Similarly, for the intelligence quoient (IQ) and behavioural (Vineland adaptive behaviour scales, VABS) scores, recorded for a subset of ASD participants (*n* = 85, *n* = 90, respectively), these scores almost all correlate and increase with age, but none correlate with direct or indirect sharing score.

## 3. Discussion

### 3.1. Psychomotor structure of play in autism

This study provides novel insights into the motor organisation of social play in young children with ASD, OND and WP, using a computational approach within an ecologically valid digital game environment. Our findings reveal that children with ASD exhibit distinct patterns in their goal-directed motor actions during pretend social food-sharing play, characterised by a developmental shift towards an indirect, two-step approach, in contrast to the more direct, single-step strategy observed in WP children. These differences in motor organisation are evident even at a young age and precede the higher-order social-cognitive and emotional regulation differences typically associated with autism, suggesting a more fundamental psychomotor component to the condition [23].

The observation that children with ASD and OND completed fewer food delivery swipes overall aligns with previous research highlighting reduced and less varied play in these groups [1–5]. However, the nuanced developmental trajectories we uncovered are particularly illuminating. While all groups demonstrated an increase in food delivery swipes with age, the plateau observed in the ASD group after 50 months suggests a potential ceiling effect in their engagement with this type of goal-directed play, contrasting with the continued development in WP and OND children. This may indicate a divergence in the developmental trajectory of motor engagement in social play in ASD, potentially impacting the learning opportunities afforded by play and their subsequent cognitive and social development [10,11]. The most striking finding was a qualitative difference in sharing strategies. The lower direct sharing scores and lack of developmental increase in this measure within the ASD group, juxtaposed with a significant increase in inter-plate swipes and indirect sharing score, point to a fundamental difference in the neuropsychological composition that underpins motor organisation in ASD children. WP children increasingly adopted a direct, efficient approach to food sharing, moving food directly from the food zone to each plate, reflecting a single-step, goal-integrated strategy. Conversely, children with ASD, particularly as they aged, demonstrated a preference for an indirect, two-step process, involving stacking food on one plate before redistributing. This suggests a sequential, rather than simultaneously integrated, approach to the game’s goal attainment in their motor organisation of play.

This indirect strategy in ASD children can be interpreted in several ways. It might reflect a difference in anticipatory motor planning, where the immediate action of stacking food precedes the distributed sharing goal. It could also indicate a different attentional focus, perhaps initially drawn to object manipulation (stacking) before the social goal of even distribution. More fundamentally, this two-step approach is probably developed from ontogenetically prior differences in prospective motor organisation [23] and its motor sequencing [17,31,32]. Regardless of the specific mechanism, the consistent emergence of this indirect pattern across the ASD group underscores a distinct motor organisation of play that diverges from typical and OND development to give a psychomotor difference in the structure of embodied autistic experience [35–37].

Reduction in the imaginative, pretence aspects of play are well documented [13] and have been a target of intervention support [36,37]. However, quantification of its underpinning motor structure and relationship to imaginative play is essential to understand the psychomotor foundation of imagination and symbolic play [16,20]. Previous studies of motor structure in autism have analysed the kinematic structure of single action units in serious game adaptations [24,27,28,38,39] and by using more traditional motor control paradigms [26,40–44]. These studies demonstrate a subtle, but significant, disruption to the prospective motor organisation of single motor actions, e.g. a single reach-to-target. Such single-action disruption leads to continuous, sub-second moment-by-moment corrections [26] that thwart prospective prediction of single units [23]. However, only a handful of studies, using prescriptive functional paradigms, have begun to measure disruption to the manner in which these are chained or sequenced [32,33,45–47]. Our analysis significantly advances the current state-of-the-art of the motor signature in autism to include new understanding of its organisation across multiple action sequences self-generated in play, and begins to show how its motor organisation impacts, or is impacted by, integrations of imagined future possibilities—the motor source of pretence and symbolic understanding [16,21].

The finding that the difference between direct and indirect sharing strategies (sharing score difference) decreases with age in WP children but increases in ASD children further emphasises this developmental divergence. WP children refine their approach towards a more efficient, direct strategy as they mature, while ASD children increasingly rely on the indirect, step-wise approach. This divergence in motor strategy may have broader implications for social learning and interaction, as the ability to efficiently and directly share goals by common patterns of action sequence is crucial in social contexts [48].

The influence of ASD severity and sex on gameplay patterns also warrants consideration. The decrease in food delivery swipes and sharing scores with increasing ASD severity reinforces the link between the observed motor differences and the core characteristics of autism. The intriguing sex-based differences, with ASD males showing age-related increases in play scores while ASD females did not, and ASD females exhibiting higher sharing scores overall, suggest potential sex-specific manifestations of motor organisation in autistic play. This aligns with growing recognition of sex-based differences in ASD presentation, and may reflect distinct underlying neurobiological pathways [49]. However, we caution against over-interpretation of these data as variable group sizes affect statistical confidence. Further investigation into these sex differences is required for a more nuanced and robust understanding of the role of sex in autistic psychomotor development.

### 3.2. Future directions

The use of a specific digital game, while ecologically valid within this context, may or may not generalise to other forms of play. Future research should explore motor organisation in other play scenarios, including free play and complex social interactions in play. The framework of second-person neuroscience suggests that such social interactions will differ depending on the individuals involved, e.g. dyads of individuals with autism differing from interactions between one person with and one person without autism [34]. Markerless motion capture and video-based pose-estimation technologies enable the digitisation of whole movement for this next step in computational analysis [50]. Furthermore, the cross-sectional design of the study prevents definitive conclusions about developmental trajectories. Longitudinal studies tracking motor play patterns from infancy through childhood are needed to fully elucidate the developmental course of these differences and their relationship to later social and cognitive outcomes.

Future research should also delve deeper into the underlying mechanisms driving these motor organisation differences in ASD. Investigating the neural correlates of these distinct play patterns using neuroimaging techniques could provide valuable insights into the brain regions and networks involved. Furthermore, exploring the role of specific motor skills, such as fine motor coordination, motor planning and sequencing abilities, in shaping play patterns would be beneficial. Finally, intervention studies designed to target motor organisation in play, and examine its impact on social communication and interaction skills in children with ASD, could have significant practical implications for early intervention and support [51,52].

## 4. Conclusion

In conclusion, this study provides evidence for distinct motor organisation patterns in the pretence play of children with ASD. The identification of an indirect, two-step pretend sharing strategy as a defining feature of autistic play, contrasted with the direct, single-step approach in typically developing children, offers a novel perspective on the psychomotor underpinnings of autism. These findings highlight the importance of considering motor organisation as an essential aspect of play development in ASD, with implications for early detection, understanding the developmental trajectory of autism and informing targeted interventions that address the fundamental motor components of social interaction and play. By shifting focus beyond solely social-cognitive aspects, we can gain a more comprehensive understanding of autism and develop more effective strategies for support and inclusion.

## 5. Methods

### 5.1. Participants

Participants were sourced from both a clinical study of the efficacy of ASD detection using tablet games [53] and the pre-trial tests used to train the diagnosis algorithms. Participants from 30 to 72 months (2.5 to 6 years) in age were included. Participants within that age range were taken from both the trial and pre-trial datasets creating three groups: (i) WP (441 participants, age range: 30–72 months, median 53 months); (ii) ASD (372 participants, age range: 30–72 months, median 54 months); and (iii) OND (64 participants, age range: 30–71 months, median 55 months). Children were recruited from preschools and diagnostic pathways in Scotland, Sweden and Poland.

Children with uncorrected hearing or visual impairments were excluded from participating. The ASD group included those with a diagnosis on the basis of the Diagnostic and Statistical Manual of Mental Disorders, 5th Edition (DSM-5), International Classification of Diseases, 10th Revision (ICD-10), Autism Diagnostic Observation Schedule, 2nd Edition (ADOS-2) or Autism Diagnostic Interview-Revised (ADI-R). Children without a diagnosis or suspicion by parents, teachers or clinicians of neurodevelopmental conditions were included in the WP group. The OND group is a heterogeneous collection of neurodevelopmental disorders, with the largest sub-groups being Down syndrome (21 participants) and language delay and disorder (22 participants). The other 14 OND participants are from various categories including a number with a primary diagnosis of global developmental delay, learning disability, mild mental retardation, cerebral palsy, VACTERL association, anxiety, attention deficits and suspected aphasia, and another seven OND participants from undefined neurodevelopmental disorders.

Ethical permission for this study was granted by the University of Strathclyde Ethics Committee, the Swedish Ethical Review Authority and an NHS West of Scotland Ethics Review Board (reference number: 17-WS-0223 377 231435). The trial registration number was NCT03438994, with the study detailed on ClinicalTrials.gov [51].

### 5.2. Data collection

This study encompasses a secondary analysis conducted on datasets collected to develop and test the accuracy of a smart tablet application for early identification of autism [52,53]. Prior to data collection, a parent or caregiver of each child signed consent for participation and permission for data use in research. Smart tablet gameplay sessions took place in school, clinical or home settings. The tablet (i.e. iPad mini) was inserted in a bumper case, and it was placed flat on a table for gameplay. Before the 5 min gameplay for data collection, a 2 min acclimatisation phase was provided to allow the children to get to know the game and develop their interest in it. Some guidance by the researcher was permissible to encourage interest.

The sharing game began with a food item being placed on the table in front of four game characters (figure 1). When touched this food is divided into four pieces, with the game demonstrating how a piece of food may be placed on the plate in front of a character. The placement of food in front of a game character elicited a celebration; following even distribution of food pieces to the four game characters, the characters exclaimed, for example, ‘Yipee!’, and then the food pieces were consumed for 3 s before the game was reset with a new food item and characters ready to be fed. The game further incentivised the even distribution by displaying characters as sad when more than one portion was placed on another character’s plate instead of theirs.

Participants were encouraged to play the food-sharing game for 5 min while their touch trajectories on the tablet screen were recorded. Age and sex information was recorded. For the ASD group, a severity level was provided for 163 of the 372 ASD participants; rated depending on the support they needed in their daily life: Level 1—requiring support (*n* = 64); Level 2—requiring substantial support (*n* = 82); Level 3—requiring very substantial support (*n* = 17).

Psychosocial measures were available for a subset of participants. For a subset of ASD participants, IQ was assessed using the Wechsler preschool & primary scale of intelligence (WPPSI), which provides general (*n* = 85) and domain-specific scores on subtests evaluating verbal comprehension, visual spatial abilities, fluid reasoning, working memory and processing speed. The VABS gives overall (*n* = 90) and domain-specific scores assessing communication, daily living skills, socialisation, motor skills and maladaptive behaviour. Observations of children’s socio-emotional status were recorded on the day of data collection (*n* = 687), including mood, arousal, cooperativity, interest in tablet games and distractibility, and were rated by the researcher and scored on a scale from 1 to 5.

### 5.3. Swipe processing

Swipes are defined to capture a user’s gameplay interactions. A swipe begins when a user makes contact with the tablet screen, and ends when that contact is removed from the screen. Multiple contacts, from using multiple fingers and/or hands, produce multiple simultaneous swipes. To track multiple swipes, each recorded contact is matched to its swipe by comparing its location with the predicted location of a swipe’s next contact. These predictions are generated by using the four previous contacts to generate a spline curve that estimates the next location. If a spline curve cannot be generated from those previous contacts, then linear interpolation is applied to predict the next touch contact location. The unassigned touch contact is then assigned to the swipe with the closest match to the *predicted* touch contact location.

### 5.4. Screen zones

All of a user’s touch contacts were recorded in the form of swipes along the tablet surface. A network is constructed from these swipes to provide insights into the activity of the participant from the aggregated pattern of their movements.

The first step to converting a participant’s touch swipes into a network was to define a number of zones in the gameplay area. Each zone was represented by a vertex in the network, with the connection weight increasing between two zones when a swipe begins in one zone and finishes in another. The defined zones were chosen to overlay key objects and areas in the sharing game. Note that each snap-to-plate zone depicted in figure 1 also contains a smaller zone centred on a plate. These plate zones include the area around the plate where food is placed and extend upwards to account for when food items are piled up on a single plate.

The movement of food items in the sharing game was inferred by tracking swipes with touch contact positions recorded to or from a plate or snap-to-plate zone (figure 1), recorded as food delivery. Limited availability of movable interactive objects and the restriction of food items to either the food or the snap-to-plate zones ensured the predominant majority of swipes beginning in these zones executed food delivery swipes.

### 5.5. Swipe metrics

To identify differences in gameplay patterns, the number of swipes that connect key zones in the game during gameplay were monitored:

— *Number of food delivery swipes*—the number of swipes that transition from the food zone to the snap-to-plate zones.
— *Number of inter-plate swipes*—the number of swipes that begin in plate zones (inside a snap-to-plate zone) and then transition to a different snap-to-plate zone.

### 5.6. Network definition

A directed graph is defined as *G* = (*V,E*), where there is a set of *V* vertices and *E* edges, which are ordered pairs. A swipe pattern network is constructed by combining a complete graph *C* with a swipe transition matrix *T* that is based on a participant’s swipe pattern. In the complete graph *C*, the vertices are all connected to each other with the same small edge weighting where $C_{ij}=0.01\forall i,j\in V$ (see electronic supplementary material for further details on this weighting). The complete graph *C* is used as a consistent baseline for each participant’s swipe pattern network. This facilitates the calculation of the sharing score, which is based on a comparison of network eigenvector entries.

**Figure 6.**
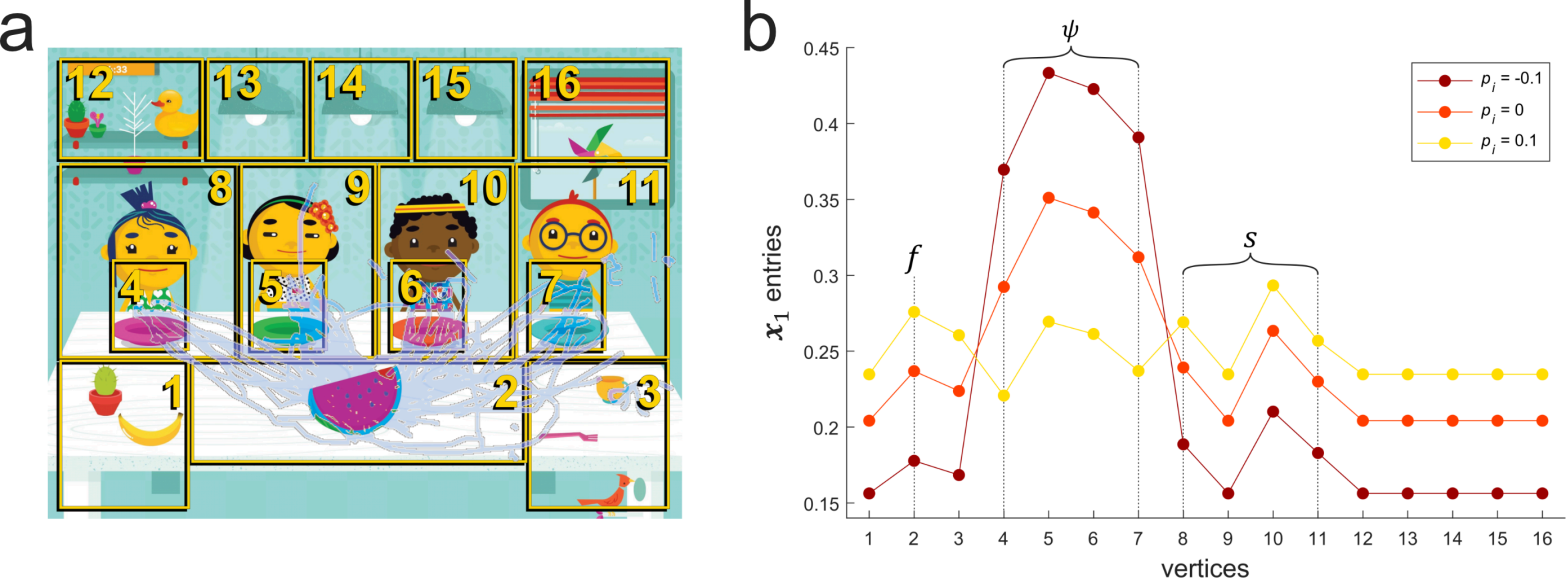
(a) The sharing game overlaid with swipes (light blue) from one WP participant with zones numbered (yellow) according to their associated vertex. (b) An eigenvector plot for this participant’s swipe pattern network, where the entries of eigenvector $x_{1}$ are displayed for different perturbation values, $p_{i}.$ The vertices corresponding to sets ψ, s and f are highlighted.

The swipe transition matrix *T* consists of 16 vertices, associated with the 16 zones displayed in figure 6. The matrix *T* is created so that the weight of each edge *T_ij_* is equal to the number of swipes, connecting an origin vertex *i* and a destination vertex *j*. The swipe transition matrix only contains swipes that transfer between zones, i.e. $T_{ii}=0\forall i\in V$, and so does not record taps on the screen or swipes that stay within a single zone. For analysis of direct food delivery, $T^{D}$ is defined such that food delivery swipes must originate from the food zone (vertex f) and end in the snap-to-plate (vertices s) zones or plate zones (vertices ψ) as defined in figure 1. All food delivery swipes whether they end in snap-to-plate or plate zones are defined such that their destination vertex *j* is in the plate zone set ψ (i.e. $T_{ij}^{D}\geq 0\forall i\in f$, where j∈ψ, else $T_{ij}^{D}=0$). When considering direct food delivery, swipes ending in a plate zone, that do not originate in the food zone, are reconnected such that the adjacent snap-to-plate zone is taken as the swipe destination. This swipe reconnection enables the graph analysis to monitor a participant’s adherence to the food-sharing gameplay narrative, as discussed in the following section, as swipes not originating in the food zone will not be moving food items (with the exception of inter-plate swipes to be incorporated shortly).

The swipe transition network is updated $A_{T}=\left(T^{D}/\sum_{i,j\in V}T_{ij}^{D}\right)$, such that the proportion of swipes transitioning between zones or remaining within a zone, with reference to the total number of swipes, are used as edge weights. The swipe pattern adjacency matrix for direct food delivery is defined as $A_{D}=A_{T}+C$ .

Swipes originating from the set of plate zones can also be responsible for food movement, referred to as inter-plate swipes (figure 1), where multiple food items can be placed on a single plate and items then transferred between plates. To account for both direct and indirect food delivery, a swipe transition matrix $T^{I}$ is defined such that food delivery swipes are allowed to originate from either food or plate zones where, regardless of whether they end in a snap-to-plate or plate zone, their destination vertex *j* is in the plate zone set ψ (i.e. $T_{ij}^{I}\geq 0\forall i\in \{f,\psi \}$, where j∈ψ, else $T_{ij}^{I}=0$). Swipes that transition from a plate zone to the adjacent and surrounding snap-to-plate zone are recorded in the swipe transition network, but their destination is kept as the snap-to-plate zone to reflect that this activity does not contribute to food sharing with the food already present on the plate it will return to upon release. The swipe pattern adjacency matrix including indirect food delivery defined as $A_{I}=\left(T^{I}/\sum_{i,j\in V}T_{ij}^{I}\right)+C$.

### 5.7. Sharing score

The game incentivises the placement of food on all four of the plates in front of the game’s characters, as described above. Therefore, a graph metric (sharing score) was created to monitor a participant’s focus on the food-sharing aspect of gameplay. This measure considers both the proportion of swipes devoted to food delivery and the relative prioritisation of food placement on the four plates. A direct and indirect sharing scores are calculated, where the methodology only differs in its use of adjacency matrix, with $A_{D}$ and $A_{I}$ used respectively.

The first left eigenvector of the adjacency matrix in this context can be interpreted as a metric for the relative popularity of zones as destinations for finger swipes [35]. The first left eigenvector $v_{1}$ of an adjacency matrix is associated with the largest eigenvalue *λ*_1_, where $v_{1}A=\lambda_{1}v_{1}$. For a directed graph, this eigenvector can be exploited to assess the popularity of a vertex as a destination for routes through a graph [54]. In the food-sharing game, a measure of destination popularity could be taken as the number of swipe arrivals in a particular game zone. The eigenvector assessment provides further insight as the eigenvector entries are a product of both edge weights (aggregated arrival numbers) and the prominence of vertices from which edges depart [35]. Therefore, able to reduce the prominence of a plate with multiple food items placed on it when those items are redistributed (see the electronic supplementary material for further details). The first eigenvector of a non-negative matrix is frequently used to determine the centrality of nodes in a graph for both directed [54] and undirected graphs [55], where it is commonly referred to as eigenvector centrality [35]. For the unperturbed swipe pattern adjacency matrix A defined here, zones associated with vertices that have the largest first left eigenvector entries are the most popular destinations for gameplay swipes. All food delivery swipes connect to the destination vertices in ψ, as described in the previous section. Therefore, the plate vertices in ψ are usually associated with the largest entries of $v_{1}$ for *A,* as they often receive the most incoming swipes during gameplay.

By monitoring the eigenvector entries of the four vertices in ψ, associated with the four game character’s plates, the relative popularity of each plate as a destination can be identified to reveal focus on food delivery relative to other gameplay activity. To evaluate the focus on even food delivery, these eigenvector entries are monitored after applying an artificial perturbation to the graph, as visualised in figure 6. This perturbation is applied to the vertices in ψ using a vector of perturbation values **p**, to produce a perturbed matrix *P*,


P=A−diag(p),

where $p_{i}=0\forall i\notin \psi$.

This perturbation alters the first left eigenvector entries. When perturbing a single vertex *i*, the effect of *p_i_* < 0 is that *x_i_ > v_i_* where $x_{1}$ and $v_{1}$ are the first eigenvectors of *P* and *A*, respectively, and ||$x_{1}$|| = ||$v_{1}$||. Conversely, the effect of *p_i_ >* 0 is that *x_i_ < v_i_*. Hence, for a connected graph, applying a large enough negative perturbation *p_i_* to a single vertex *i* ensures that *x_i_* = max($x_{1}$) and, conversely, a large enough positive *p_i_* ensures that $x_{i}\neq max\left(x_{1}\right).$

When applying the same perturbation *p_i_* to all vertices in ψ, a large enough negative perturbation ensures that the largest four entries of $x_{1}$ are associated with vertices in ψ—as shown in figure 6 for $p_{i}$ = −0.1. Conversely, a large enough positive perturbation ensures that the largest four entries of $x_{1}$ are not associated with any of the vertices in ψ. In figure 6, increasing $p_{i}$ is shown to result in decreasing eigenvector ($x_{1}$) entries for vertices 4 to 7 in ψ. For $p_{i}$ = 0.1, only vertex 5 is clearly among the top four entries in magnitude, therefore the sharing score lies between 0 > *p_i_* > 0.1 (see the electronic supplementary material for further examples).

The *sharing score* is defined for each participant as the largest perturbation *p_i_* for which the largest four entries of $x_{1}$ are associated with those vertices in ψ. The sharing score rewards devotion to the even food delivery aspect of gameplay, as either an uneven distribution of food delivery swipes or the presence of swipes unrelated to food delivery will decrease the largest perturbation *p_i_*. The first eigenvector, $x_{1}$, is assessed from the aggregated pattern of swipes over the gameplay session, and does not consider the patterns during each cycle of food distribution before the game resets food items on the table. Normalising the edge weighting to represent the proportion of swipes further distinguishes this analysis from the assessment of *number of food delivery swipes*.

### 5.8. Statistical methods

The non-parametric Wilcoxon rank sum test is applied throughout on pairwise distribution comparisons, where the distributions often do not meet the normality assumption. The Wilcoxon rank sum test investigates equality of population medians of two independent samples X and Y, by considering the number of times *y* precedes an *x* in an ordered arrangement of the elements. In this way, it tests whether one variable tends to have values higher than the other. When the distributions are equal this test compares the difference in medians, but can also detect differences in distribution shape even when medians are similar [56].

For determining the significance of the monotonic—but not always linear—correlations with age, Spearman’s rank correlation coefficient is employed.

### 5.9. Boxplot visualisations

The boxplots are presented with a notch—tapered region—above and below each median line. The notches provide a visual indication of significant differences, where non-overlapping notches have different medians at the 5% significance level. This is based on a normal distribution assumption, but the median comparison is stated to be reasonably robust for other distributions [57].

## Data Availability

All derived data, swipe visualisations and swipe pattern networks, are made available on Zenodo [58]. There are restrictions on detailed participant data due to privacy and confidentiality. Contact the Research Data Management & Sharing at the University of Strathclyde (researchdataproject@strath.ac.uk) for further information about the data and conditions for access.
